# Chemical Profiling and Antimicrobial Properties of Honey Bee (*Apis mellifera* L.) Venom

**DOI:** 10.3390/molecules26103049

**Published:** 2021-05-20

**Authors:** Irina Tanuwidjaja, Lidija Svečnjak, Domenika Gugić, Marko Levanić, Slaven Jurić, Marko Vinceković, Mirna Mrkonjić Fuka

**Affiliations:** 1 Department of Microbiology, Faculty of Agriculture, University of Zagreb, 10000 Zagreb, Croatia; ianuwidjaja@agr.hr (I.T.); domenika.gugic@gmail.com (D.G.); mfuka@agr.hr (M.M.F.); 2 Department of Fisheries, Apiculture, Wildlife Management and Special Zoology, Faculty of Agriculture, University of Zagreb, 10000 Zagreb, Croatia; markolevanic95@gmail.com; 3 Department of Chemistry, Faculty of Agriculture, University of Zagreb, 10000 Zagreb, Croatia; sjuric@agr.hr (S.J.); mvincekovic@agr.hr (M.V.)

**Keywords:** honey bee venom, melittin, total protein, FTIR-ATR spectral profile, antibacterial activity, conventional antibiotics

## Abstract

The incidence of antibiotic resistance in pathogenic bacteria has become an alarming clinical and social problem. Therefore, the demand for alternative antimicrobial compounds has increased. In this study, a chemical profile of honey bee (*Apis mellifera* L.) venom (HBV) has been determined by HPLC and FTIR-ATR spectroscopy, and tested for antibacterial activity, as well as efficiency with regard to conventional antibiotics. The investigated HBV was of high quality with melittin and total protein contents of 70.10 ± 7.01%, and 84.44 ± 3.12 g/100 g, respectively. The purity of HBV was confirmed by FTIR-ATR spectral profiling, which revealed a unique pattern of absorption bands that are characteristic of its major fractions. In addition, HBV showed a broad spectrum of activity against all three tested biomasses of potentially pathogenic Gram-positive and Gram-negative bacteria with MIC values ranging between 12.5 and 200 µg/mL, and MBC between 12.5 and 400 µg/mL. When compared to conventional antibiotics, HBV (400 µg) showed up to 27.8% efficiency of tetracycline (30 µg), 52.2% erythromycin (15 µg), 21.2% ciprofloxacin (5 µg), and 34.6% of ampicillin-sulbactam (20 µg). The overall results demonstrate the therapeutic potential of the analyzed HBV.

## 1. Introduction

Since the discovery of antibiotics, their uncritical application and overuse have led to the development of antimicrobial drug resistance in numerous bacterial pathogens. Such bacteria are becoming a serious clinical and social problem throughout the world. Thus, new effective antibacterial agents with a novel mode of action need to be developed [1]. Antimicrobial peptides (AMPs), the defense peptides found in different hosts, are one such attractive therapeutic candidate which can kill a broad range of bacteria by disrupting their membranes. In addition, bacteria do not tend to develop drug resistance to AMPs [2,3].

Natural products such as venoms from different animals (e.g., bees, wasps, snakes, and scorpions) are an important source of such new bioactive peptides [4,5] and promising antimicrobial agents against various microbial pathogens [5,6]. Honey bee (*Apis mellifera* L.) venom (HBV), secreted in honey bee workers’ venom glands as a protection mechanism, has long been used to treat an array of medical conditions [7,8]. It is a complex mixture of substances (primarily polypeptides and enzymes) exhibiting a wide range of biological activities, including antimicrobial, cytotoxic, hemolytic and anti-inflammatory activity [9,10,11,12]. Generally, the biological activity of HBV stems from peptides (melittin, apamin, adolapin, the mast cell degranulating peptide, secapin, procamine, protease inhibitor, tertiapin, and other small peptides), enzymes (phospholipase A2 (PLA2), phospholipase B, hyaluronidase, phosphatase, α-glucosidase, acid phosphomonoesterase, and lysophospholipase), amines (histamine, dopamine, and noradrenaline), amino acids (aminobutyric acid and α-amino acids), sugars (glucose and fructose), phospholipids, and volatile compounds [13,14,15,16]. However, the purity and composition of HBV, as well as the concentration of its substances, differ significantly [17,18].

The major compound and the principal bioactive peptide with antimicrobial activity in HBV is mellitin, which comprises up to 60% of the dry HBV weight [19]. The antimicrobial activity of pure mellitin or natural HBV mix, against several Gram-positive and Gram-negative bacteria, is well documented and the effect varies with different HBV or different species applied [11,20,21,22]. Although the effect of any antimicrobial compound is dependent on the initial microbial density (cells per volume unit), the majority of studies primarily focus on testing the effect of HBV on a single microbial biomass. For example, it is well-known, for antibiotics, that the relative amount of drug needed to inhibit the growth of a bacterial population increases with the density of that population and, for some antibiotics, the minimum inhibitory concentration (MIC) changes with the density of bacteria exposed [23,24]. In addition, depending on the microorganism whose antimicrobial susceptibility is tested, different antibiotic concentrations are used. In addition, considering the increasing prevalence and complexity of antimicrobial resistance mechanisms, the guidelines for antimicrobial susceptibility testing are prone to modifications due to the changes in MIC breakpoints and the detection of new resistance mechanisms [25,26].

Therefore, the objective of this study was to evaluate the antibacterial activity of honey bee venom against three different biomass quantities (3, 6 and 8 log CFU/mL) of selected potentially pathogenic Gram-positive (*Staphylococcus aureus*, *Listeria innocua*, and *Bacillus cereus*), and Gram-negative (*Escherichia coli* and *Salmonella enterica*) bacteria by determining the minimum inhibitory (MIC) and bactericidal (MBC) concentrations, and compare it with the conventional antibiotics. In addition, we aimed to determine the quality and purity of the HBV sample prior to microbiological assay by performing a detailed chemical profiling using classical, chromatographic and spectroscopic analytical methods.

## 2. Results and Discussion

### 2.1. Honey Bee Venom Composition

The results of HPLC analysis and the Kjeldahl method confirmed the good quality of the investigated HBV sample, where the melittin and total protein contents were 70.10 ± 7.01%, and 84.44 ± 3.12 g/100 g, respectively. Compared to most of the reports on the HBV, where the melittin content ranged from 40 to 60% [8,19,27,28], with an average of 50%, and the total protein content from 47.2% to 77.8% [29,30,31], the analyzed sample showed above-average high-quality properties.

Melittin and phospholipase A2 (PLA2) are not only the most abundant and the most studied compounds of honey bee venom [8]; they are also considered to be responsible for most of the beneficial effects of venom [16], including the antimicrobial activity against various microorganisms [32,33,34,35]. The quantitative analyses of selected bee venom components (e.g., melittin, dopamine, and histamine) have been an indirect means of measuring its purity and quality, especially given that the quality can be strongly affected by the venom storage, which may cause degradation of the venom components (e.g., by autolysis due to presence of proteases in the venom) [36]. The purity of bee venom is, nowadays, mostly determined based on the melittin content.

### 2.2. Spectral Assignment of Molecular Vibrations Observed in Bee Venom Powder/FTIR-ATR Spectral Profiling

**Figure 1:**
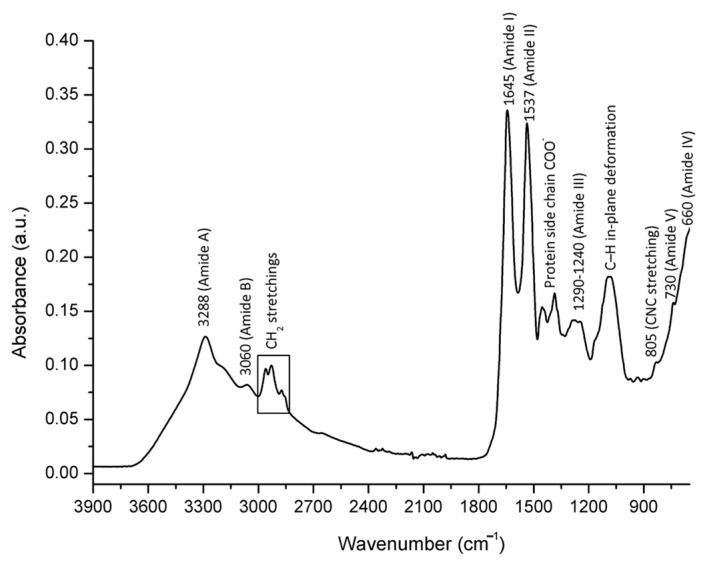
Characteristic FTIR-ATR spectrum of dried honey bee venom (powder) with the assignation of major underlying molecular vibrations.

The results of the spectral analysis revealed a characteristic FTIR-ATR spectrum of honey bee venom powder distinguished by a unique pattern of absorption bands primarily arising from molecular vibrations related to the structure of honey bee venom’s major fractions—peptides and proteins (Figure 1). Given that dried honey bee venom represents a complex biological mixture of various polymeric macromolecules with predominant peptide and protein (enzyme) constituents, such as the most abundant 26-amino-acid polypeptide melittin (comprising ~40–60% of the dry weight of bee venom) and PLA2 (~10–12%) [15,27,37], the absorption bands of these macromolecules predominate in its IR spectrum. As presented in Figure 1, the FTIR-ATR spectrum of HBV powder is characterized by a broad medium-intensity absorption band observed in the spectral range from 3500 to 3100 cm^−1^ (with an absorption maximum at 3288 cm^−1^), which is assigned to the N–H stretching vibrations (amide A band) of the peptide and protein secondary structures [38,39], while low-intensity signal belonging to the same vibrational mode appears at 3060 cm^−1^ (amide B). The latter may also indicate an overtone of the amide II band [38]. Medium intensity absorptions observed at 2960 cm^−1^ and 2928 cm^−1^ correspond to CH_2_ asymmetric stretching vibrations, and they are followed by the lower intensity analyte signals at 2873 cm^−1^ and 2855 cm^−1^ that are attributed to CH_2_ symmetric stretchings.

The spectral region between 1700 and 700 cm^−1^ (fingerprint region) is populated by a series of absorption bands that are unique to the peptide/protein secondary structure. The most prominent vibrations in this region, occurring at 1645 cm^−1^ and 1537 cm^−1^, are assigned to the amide I (C=O stretching) and amide II (N–H bending and C–N stretching vibrations) bands, respectively. This is in compliance with the report on the basic spectral features of the bee venom provided by Park et al. [40]. Other amide bands are represented in the following frequencies: The peaks arising at 1454 and 1386 cm^−1^ are likely to result from the protein side chain COO^−^. A broad medium intensity absorption occurs between 1290 and 1240 cm^−1^ due to the amide III band, which comprises 30% of C–N stretching, 30% of N–H bending, 10% of C–O stretching, and 10% of O=C–N bending vibrations, while the rest belongs to other vibrations [38]. A medium intensity absorption observed at 1088 cm^−1^ can be attributed to the C–H in-plane deformation vibration of various aromatic structures in the bee venom. A weak analyte signal occurs nearby at 805 cm^−1^ due to the symmetric CNC stretching vibration of proteins (enzymes). Amide IV (primarily O=C–N bending) and amide V (N–H bending) bands have been observed at 660 cm^−1^ and 730 cm^−1^, respectively.

**Figure 2:**
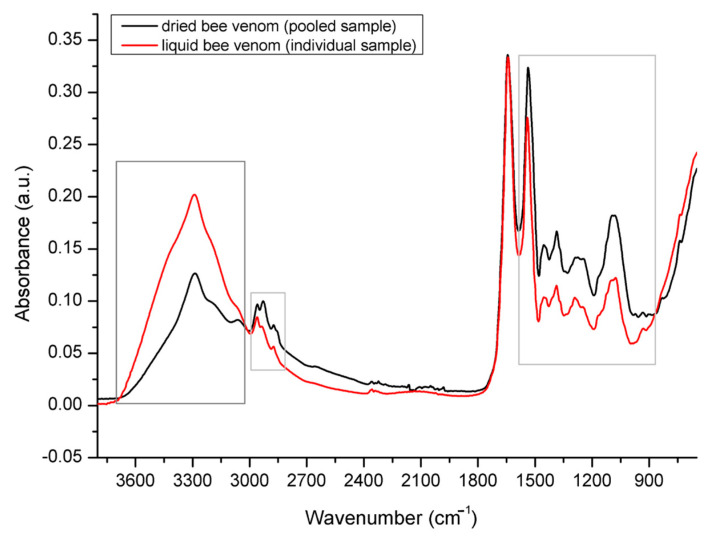
Comparative spectral features of dried vs. liquid honey bee venom (extracted from a 24-day old worker bee).

Figure 2 shows comparative spectral features of the investigated HBV powder vs. genuine liquid bee venom extracted from a 24-day old honey bee. The results have revealed similar integral spectral features of dried and liquid venom with differences exhibited in the relative absorption intensities of water and peptide/protein bands. These effects were reflected in the lower absorption intensities of the O–H stretching vibrations of water (intense absorption in the 3700–3000 cm^−1^ region) in the dried form of HBV compared to a liquid form of genuine bee venom, and an overlapping of the H–O–H deformation of water with the amide I band at 1645 cm^−1^. This was followed by higher absorption intensities of the analyte signals of other peptide/protein-based venom components observed in dried HBV. Given that no analyte signals other than those specific for HBV proteins were observed, these results confirmed the purity of dried bee venom used for the microbiological assay.

### 2.3. Antibacterial Activity of Honey Bee Venom (HBV)

The antibacterial activity of HBV was determined by evaluating minimum inhibitory (MIC) and minimum bactericidal (MBC) concentrations against different biomass quantities (3, 6, and 8 log CFU/mL) of selected Gram-positive and Gram-negative bacteria. The MIC values were calculated as the concentrations that inhibited more than 95%, and MBC more than 99%, of bacterial growth. The results are summarized in Table 1.

Both MIC and MBC values correlate with the initial biomass of the pathogen, where, with an increase in bacterial density, higher concentrations of HBV were needed for bacteriostatic and bactericidal effects. At lower and medium initial biomass levels, similar HBV concentrations (3 log CFU/mL: 12.5 µg/mL; 6 log CFU/mL: 12.5–25.0 µg/mL) suppressed the growth or completely inhibited (3 log CFU/mL: 12.5–50.0 µg/mL; 6 log CFU/mL–25.0–50.0 µg/mL) all tested pathogens. When biomass was extremely high (8 log CFU/mL), high HBV concentrations were needed to inhibit (25.0–200.0 µg/mL) or to completely kill (50.0–400.0 µg/mL) all tested bacterial strains.

In the studies of Oren and Shai [41], Han et al. [32], and Ebbensgaard et al. [42], lower or higher MIC values were reported compared to the results demonstrated in this study. However, in the aforementioned papers, the antibacterial activity of pure mellitin was predominantly tested, which makes it difficult to compare it with our results. For example, Oren and Shai [39], showed that mellitin inhibited the growth of *E. coli* at 5 µM, and different *Bacillus* species at 0.3–0.4 µM, while Ebbensgaard et al. [42] showed that mellitin inhibited *E. coli* at 16 µg/mL, and both *S. aureus* and *L. monocytogenes* at 2–4 µg/mL. On the contrary, Ebbensgaard et al. [42] determined a higher MIC of mellitin for *Salmonella enterica* (32–64 µg/mL).

Contrary to our study, Hegazi et al. [11] reported much higher MIC values of whole honey bee venom varying from 1600 to 3600 µg/mL for *S. aureus*, and from 1800 to 3800 µg/mL for *E. coli,* while Han et al. [22], on the other hand, detected much lower MIC values of whole honey bee venom, ranging from 0.085 to 0.11 µg/mL for methicillin-resistant *S. aureus*. Such differences can be attributed to the extraction method and additional venom purification, resulting in different HBV purity and mellitin content values. For example, while Hegazi et al. [11] obtained honey bee venom either by homogenization of the bee venom sac in ethanol or whole bees in saline solution, we collected the HBV by scraping the dried HBV from a glass plate covered with plastic foil, following the electro-stimulation method, which may have directly increased the HBV purity in our study. Unlike our study, Han et al. [22] further purified bee venom before the antimicrobial activity testing, which may have led to the concentration of bioactive compounds, such as mellitin, in whole honey bee venom.

Similarly to our study, Han et al. [32] investigated the antimicrobial activity of whole HBV where MIC for *E. coli* was 0.25 µg/mL and for *S. aureus* 0.06 µg/mL. On the contrary, Al-Ani et al. [34] detected a much higher MIC of whole HBV for *S. aureus* (10–60 µg/mL) and *E. coli* (60–200 µg/mL). Such differences could be explained by different bacterial species and strains, and different initial biomass used. Although our results are in line with previous studies as the lowest MIC value in our study was 12.5 μg/mL, to increase the resolution, especially at the lower range, HBV concentrations < 12.5 μg/mL should be tested.

Furthermore, in the case when bacterial growth was detected in our study only in the control wells after 24 h, the incubation was prolonged, and MIC and MBC values were determined after 48 h. The growth of *L. innocua*, *S. enterica* subsp. *enterica* and *E. coli* treated with HBV could only be detected after 48h; thus, the true HBV killing potential could be determined only after prolonged incubation. *L. innocua*, *S. enterica* subsp. *enterica* and *E. coli* are known for forming biofilms [43,44], which provide certain survival advantages. In addition to the development of complex nutrient and oxygen gradients, biofilms often contain extracellular enzymes that are important for microbial cell nutrition, thus allowing the transfer of molecules involved in cellular communication. In addition, they protect microbial cells from adverse physical (e.g., dehydration) and chemical factors, such as substances with antimicrobial activity [45].

Generally, the MBC values for all tested pathogens were either the same or double when compared to MIC values, except for *B. cereus*, most likely due to its ability to sporulate [46] and survive adverse conditions.

### 2.4. Comparison of Antibacterial Activity of HBV and Conventional Antibiotics

Antibacterial activity of HBV (100–400 µg) and conventional antibiotics (tetracycline (TE 30 µg) and erythromycin (E 15 µg) for Gram-positive bacteria, and ciprofloxacin (CIP 5 µg) and ampicillin-sulbactam (SAM 10 µg + 10 µg) for Gram-negative bacteria) was determined by the disc diffusion method. The sensitivity of all pathogens to HBV was achieved at 100 µg for Gram-negative and 300 µg for Gram-positive bacteria. Increased concentrations of HBV, up to 400 µg, were applied in order to achieve saturation. The results are summarized in Table 2.

All pathogens were sensitive to conventional antibiotics and their sensitivity to HBV increased with the HBV concentration. Only minor differences were observed between 300 and 400 µg of HBV, indicating that saturation in HBV concentration was achieved. At HBV concentrations of 300 µg, 29.1 ± 0.4% TE efficiency and 33.4 ± 0.1% E efficiency were reached when applied to *S. aureus*; in addition, 25.5 ± 1.7% TE efficiency and 47.8 ± 1.0% E efficiency towards *B. cereus*, and 19.9 ± 0.2% TE efficiency and 21.4 ± 0.3% E efficiency towards *L. innocua*, were achieved.

At HBV concentrations of 100 µg, *E. coli* was more sensitive to HBV than *S. enterica*. More specifically, at HBV concentrations of 100 µg, 15.3 ± 0.3% CIP and 25.0 ± 0.0% SAM efficiency when applied to *E. coli*, and 13.8 ± 1.8% CIP and 22.6 ± 2.7% SAM towards *S. enterica*, was reached.

The disc diffusion method showed that *S. aureus* was the most sensitive to the HBV. In general, Gram-positive pathogens were found to be more sensitive to HBV than Gram-negative, [11,47,48]. Due to the differences in the cell wall structure, mellitin, the main component of bee venom, can more easily penetrate the peptidoglycan layer of the Gram-positive bacteria [32,35,49]. The detected differences in HBV antibacterial activity may arise due to differences in methodology, the effect of inoculum, and the stability of antimicrobial compounds [50].

Furthermore, the antimicrobial activity of HBV at the highest tested concentration of 400 µg showed a considerable efficiency of conventional antibiotics. For example, HBV showed up to 27.8% TE and 52.2% E efficiency when applied to Gram-positive bacteria, and up to 21.2% CIP and 34.6% SAM in Gram-negative bacteria. Some studies have revealed that the antimicrobial activity of HBV increases when applied in combination with conventional antibiotics [22,34], where HBV interacts with cell membranes and forms ion channels, thus altering the permeability of the cytoplasmic membrane. In this way, HBV can simultaneously allow other substances, such as antibiotics, to enter the cell and cause cell lysis [51].

## 3. Materials and Methods

### 3.1. Honey Bee Venom Collection

Honey bee venom was obtained directly from the primary producer with an apiary situated in Bjelovar-Bilogora County (Croatia) during 2018. HBV was collected from 100 Carniolan honey bee (*Apis mellifera carnica* Pollman, 1879) colonies (pooled sample) by an electro-stimulation method. An internal bee venom collector, placed at the top of the 10-frame Langstroth hives under a dome, was powered by a 12-volt battery. Honey bees were stimulated with electric impulses (12 V) for 10 s followed by 10 s pauses. During the electro-stimulation, the bee workers stung the glass plates through a plastic foil. Venom was left to dry at 27 °C and a relative humidity of 50% for 35 min and collected by scraping the dried HBV (powder) from the glass slide of the venom collector using a sharp scraper. Dried HBV was stored in the dark and dry place at 4 °C prior to further analyses.

### 3.2. Chemical Characterization of Honey Bee Venom

Detailed chemical profiling of collected *A. mellifera* venom sample was carried out to determine the content of targeted bioactive compounds and to ensure the purity of the sample prior to further microbiological assay.

#### 3.2.1. Determination of Melittin in Honey Bee Venom Using High-Performance Liquid Chromatography (HPLC)

A modified version of the reversed-phase chromatography method of Rybak-Chmielewska and Szczęsna [52] was applied for the determination of the melittin content in the honey bee venom using an HPLC UV detector (Agilent 1200, Palo Alto, CA, USA). Chromatographic separation was achieved using a C18 HPLC column, linear gradient elution (B 5–80% 40 min) using two different mobile phases: mobile phase A was 0.1% trifluoroacetic acid in water, and mobile phase B was 0.1% trifluoroacetic acid in 80% acetonitrile (flow rate: 2.0 mL/min; column temperature: 25 °C). The chromatographic detection of melittin was achieved with a UV detector at 220 nm. The amount of melittin in the sample was determined by the external standard method using the calibration curve (*k* > 0.999), and the result was expressed in percentage (%). The standards were supplied by Sigma-Aldrich. The analysis was performed in triplicate and the results are expressed as mean values with standard deviation.

#### 3.2.2. Determination of Total Proteins in Honey Bee Venom

Protein (nitrogen) content was determined according to the standard ISO 1871:2009 [53] (Kjeldahl method using Kjeltec TM 2100, FOSS (Haganäs, Sweden)). A quantity of 0.5000 g of venom was weighed in Kjeldahl digestion tubes with 5 g of Kjeldahl catalyst tablets. In addition, 12 mL of concentrated sulfuric acid (97%) was added to the tubes. The suspension was carefully mixed and left overnight. Digestion tubes were transferred to the digestion unit for mineralization under vacuum. The temperature was gradually increased and the process was finished when the solution became clear and light green with no visible particles. Tubes were transferred and cooled to room temperature. Afterward, 80 mL of distilled water was added and tubes with samples were put into the Kjeltec system. Ammonia uptake after distillation was performed in an Erlenmeyer flask containing 25 mL of 4% boric acid with an indicator. The alkaline solution was dosed in five 10 mL portions and the distillation took place automatically for 4 min. Due to the presence of ammonia, the distillate turns green over time. The burette was loaded with 0.1 mol dm^−3^ HCl (volumetric standard) and used for titration of the flask content after distillation in a Kjeltec system. At the endpoint of the titration, the color of the solution became pale pink. The volume of hydrochloric acid for the titration of the sample was recorded. The analysis was performed in triplicate and the results were expressed as mean values with standard deviation. Nitrogen content (%) was calculated according to the Equation (1):

(1) $$\%N=\frac{\left[\left(a-b\right)\times c_{acid}\times f_{acid}\times 1.4007\right]}{m_{sample}}$$

where *a* is the volume of HCl spent for titration of the sample (mL), *b* is the volume of HCl spent for titration of blank (mL), *c* is the concentration (mol/L), *f* is the acid factor, and *m* is the sample weight (g).

#### 3.2.3. Chemical Fingerprinting by FTIR-ATR Spectroscopy

In addition to the analytical methods applied for quantifying major peptide/protein fractions, the sample was analyzed by infrared (IR) spectroscopy in order to provide an insight into the total chemical composition and ensure the purity of investigated HBV sample.

Dried HBV sample (powder) was analyzed by Fourier transform infrared spectroscopy (FTIR) coupled with the Attenuated Total Reflectance (ATR) recording technique to provide an insight into its total chemical composition. Infrared (IR) spectra of the bee venom sample were acquired using the Cary 660 Fourier FTIR spectrometer (Agilent Technologies, Palo Alto, CA, USA) and the Golden Gate single-reflection diamond ATR accessory (Specac). The sample was analyzed as obtained (it was only pulverized into a finer powder with a porcelain mortar). Approximately 50 mg of bee venom powder was used to acquire the spectra of a thin uniform layer of the sample. This was achieved by pressuring the bee venom powder on a diamond ATR plate using a self-leveling sapphire anvil. Absorption spectra were recorded in a mid-infrared region (4000–400 cm^−1^) using a nominal resolution of 4 cm^−1^ (at 23 ± 2 °C). Two replicate spectra of the sample (32 scans per spectrum) were recorded using different aliquots. Raw spectral data were stored and pre-analyzed using the Agilent Resolutions Pro version 5.3.0 software package (Agilent Technologies, Palo Alto, CA, USA), and further spectral data analysis and processing were carried out using Origin version 8.1 (Origin Lab Corporation). An assignation of molecular vibrations observed in the bee venom spectrum was performed using electronic spectral libraries (NIST, IMB Jena Image Library of Biological Macromolecules, Agilent Technologies, Palo Alto, CA, USA) in combination with venom-relating spectral data from the available scientific literature.

To ensure the purity of the investigated bee venom sample (absence of hive-originating impurities/particles, such as beeswax, propolis, bee pollen, or nectar), the spectra of analyzed bee venom powder were compared with a spectrum of genuine bee venom sample collected directly from a 24-day old worker bee (forager) in a liquid form using a 10 µL microcapillary tube sting stimulation (internal spectral collection of authentic bee venom stored at Laboratory for bee products analysis and bee biology, University of Zagreb Faculty of Agriculture).

### 3.3. Evaluation of Bee Venoms’ Antibacterial Activity

#### 3.3.1. Bacterial Strains

The bacterial strains used in this study were *S. aureus* subsp. *aureus* (DSM 20231), *B. cereus* (DSM 6791) and *S. enterica* subsp. *enterica* (DSM 14221) from the German Collection of Microorganisms and Cell Cultures, and *L. innocua* (ATCC 3309) and *E. coli* (ATCC 25922) from American Type Culture Collection. All isolates were stored as glycerol (25%) culture at −20 °C in the Department of Microbiology, University of Zagreb Faculty of Agriculture.

#### 3.3.2. Determination of Minimum Inhibitory (MIC) and Bactericidal (MBC) Concentrations

The minimum inhibitory (MIC) and minimum bactericidal (MBC) concentrations of HBV were determined by broth microdilution according to the Clinical and Laboratory Standards Institute reference method M27-A3 [54] in 96-well microtiter plates (Sigma-Aldrich, Taufkirchen, Germany). MIC and MBC were determined for three different cell densities (3, 6, and 8 log CFU/mL) for all tested pathogens. Several colonies of pure culture were inoculated in 10 mL of sterile saline solution (0.85%) under sterile conditions until a cell concentration corresponding to the 0.5 McFarland standard (1.5 × 10^8^ CFU/mL) was achieved. Cell suspensions were then diluted in sterile saline solution (0.85%) to obtain target biomass.

HBV was dissolved in distilled water and then filtered through a membrane filter (0.22 µm pore size, Millipore, Billerica, MA, USA) to obtain a working solution of HBV (1000 µg/mL). HBV was serially diluted two-fold in Mueller–Hinton broth in microtiter plates, to test the HBV concentration range 12.5–800 µg/mL. All tested pathogens (3, 6, and 8 log CFU/mL) were incubated with two-fold serial dilutions of HBV, at 37 °C for 24 or 48 h under constant agitation (90 rpm/min; Orbital Shaker-Incubator ES-20, Biosan, Latvia). The experiment was performed in triplicates. The bacterial growth was measured by the spectrophotometric assay as turbidity at 660 nm wavelength (EL80, BioTek Instruments, VT, USA). The bacterial growth inhibition was calculated according to Equation (2):

(2) $$\mathrm{Growth}\;\mathrm{inhibition}\;\left[\%\right]=100\times \left[1-\left(\frac{A_{t}-A_{0}}{A_{\mathrm{ct}}-A_{c0}}\right)\right]$$

where A_t_ is the absorbance of MH + HBV + bacterial suspension measured after 24 or 48 h, A_0_ the absorbance of MH + HBV + bacterial suspension measured at the beginning of the experiment (0 h), A_ct_ the absorbance of MH + bacterial suspension measured after 24 or 48 h, and A_c0_ the absorbance of MH + bacterial suspension measured at the beginning of the experiment (0 h).

#### 3.3.3. Antibacterial Assay of Bee Venom and Conventional Antibiotics

The antibacterial effects of HBV and conventional antibiotics were tested by using disc diffusion assays [55,56]. Pure cultures were prepared by culturing the test strains in 1.5 mL of brain heart infusion broth (BHI broth, Biolife, Italy). After the incubation at 30 °C for 24 h, the test strains were streaked on BHI agar plates and incubated for an additional 24 h, in order to obtain a pure culture. Several colonies were taken with a sterile loop and inoculated in 10 mL of sterile saline solution (0.85%) at a cell concentration corresponding to 0.5 McFarland standard (1.5 × 10^8^ CFU/mL). A sterile cotton swab was used for spreading diluted cell suspension on Mueller–Hinton (MH, Biolife, Italy) agar plates. Sterile blank paper discs (7-mm diameter, Biorad, France) were then placed on the MH agar’s surface, and HBV (100 µg, 200 µg, 300 µg, and 400 µg) was added to each disc. Three replicates were made for each treatment. The results were presented as a mean inhibition zone, including the diameter of the disc, in mm.

Antibiogram disks including tetracycline (TE; 30 µg) and erythromycin (E; 15 µg) for Gram-positive isolates, and ciprofloxacin (CIP; 5 µg) and ampicillin-sulbactam (SAM; 10 µg + 10 µg) for Gram-negative isolates, were added. The plates were incubated at 37 °C for 24 h, and the zones of inhibition were measured. The results were calculated as mean inhibition zone in mm, and as a percentage (%) of antimicrobial activity compared to the antimicrobial activity of tetracycline and erythromycin for Gram-positive isolates, and ciprofloxacin and ampicillin-sulbactam for Gram-negative isolates.

### 3.4. Statistical Analysis

All data were expressed as mean values ± standard deviation of the mean (SD). Statistical differences between groups were determined using Analysis of Variance (ANOVA) followed by the post-hoc Tukey HSD test. Differences with *p* < 0.01 were considered significant. All statistical analyses were performed in R environment version 3.0.2 [57].

## 4. Conclusions

The chemical composition, purity, and antibacterial activity of honey bee venom (HBV) obtained directly from the primary producer were investigated in this study. The analyzed HBV showed above-average high-quality properties with melittin and total protein contents of 70.10 ± 7.01%, and 84.44 ± 3.12 g/100 g, respectively. The results of the spectral analysis revealed a characteristic FTIR-ATR spectrum of HBV powder distinguished by a unique pattern of absorption bands primarily arising from molecular vibrations related to the major fractions of HBV—peptides and proteins. Besides high melittin and protein content, the broad spectrum of antibacterial activity of the investigated HBV against all tested potentially pathogenic Gram-positive and Gram-negative bacteria (*Staphylococcus aureus*, *Listeria innocua*, *Bacillus cereus*, *Escherichia coli* and *Salmonella enterica*) was observed, irrespective of the bacterial biomass applied (3, 6 or 8 log CFU/mL). The MIC values ranged between 12.5 and 200 µg/mL (3 log CFU/mL: 12.5 µg/mL; 6 log CFU/mL: 12.5–25.0 µg/mL; 8 log CFU/mL: 25–200 µg/mL), and MBC values ranged between 12.5 and 400 µg/mL (3 log CFU/mL: 12.5–50.0 µg/mL; 6 log CFU/mL: 25.0–50.0 µg/mL; 8 log CFU/mL: 50.0–400 µg/mL). When compared to conventional antibiotics, HBV (400 µg) showed up to 27.8% efficiency of tetracycline (30 µg), 52.2% erythromycin (15 µg), 21.2% ciprofloxacin (5 µg), and 34.6% of ampicillin-sulbactam (20 µg). The results presented in this study revealed above-average high-quality properties of investigated honey bee venom, and indicate that HBV is a good source of alternative antimicrobial compounds.

## Figures and Tables

**Table 1:** Minimum inhibitory (MIC) and minimum bactericidal (MBC) concentrations against different biomass quantities of selected bacterial strains.

Bacterial Strains	MIC/MBC [µg/mL]		
	3 log CFU/mL	6 log CFU/mL	8 log CFU/mL
*S. aureus* subsp. *aureus*	12.5/12.5	25.0/50.0	200.0/400.0
*B. cereus*	12.5/50.0	12.5/50.0	25.0/50.0
*L. innocua*	12.5/25.0	12.5/50.0	25.0/100.0
*E. coli*	12.5/25.0	25.0/25.0	200.0/400.0
*S. enterica* subsp. *enterica*	12.5/25.0	25.0/50.0	200.0/200.0

**Table 2:** In vitro antibacterial activity of HBV and conventional antibiotics as shown by disc diffusion method. Each result is presented as a mean inhibition zone in mm ± standard deviation (*n* = 3). Statistical differences were determined by analysis of variance (ANOVA) and the post-hoc Tukey HSD test.

Bacterial Strains	HBV (100 µg)	HBV (200 µg)	HBV (300 µg)	HBV (400 µg)	TE (30 µg)	E (15 µg)	CIP (5 µg)	SAM (20 µg)
*S. aureus* subsp. *aureus*	8.5 ± 0.7 ^afgh^	9.6 ± 0.1 ^b^	9.6 ± 0.1 ^bg^	9.6 ± 0.1 ^bg^	33.0 ± 0.0	28.8 ± 0.4	n.d.	n.d.
*B. cereus*	6.0 ± 0.0 ^d^	7.0 ± 0.0 ^e^	8.0 ± 0.0 ^af^	8.8 ± 0.4 ^afgh^	31.5 ± 2.1	16.8 ± 0.4	n.d.	n.d.
*L. innocua*	0.0 ± 0.0 ^c^	0.0 ± 0.0 ^c^	8.3 ± 0.4 ^afgh^	8.5 ± 0.1 ^afgh^	41.5 ± 2.1	38.5 ± 2.1	n.d.	n.d.
*E. coli*	6.5 ± 0.0 ^de^	8.0 ± 0.0 ^f^	9.0 ± 0.0 ^g^	9.0 ± 0.0 ^abgh^	n.d.	n.d.	42.5 ± 0.7	26.0 ± 0.0
*S. enterica* subsp. *enterica*	6.3 ± 0.4 ^de^	6.0 ± 0.0 ^d^	8.5 ± 0.0 ^h^	9.0 ± 0.0 ^bh^	n.d.	n.d.	45.5 ± 0.3	27.8 ± 1.8

HBV—honey bee venom; TE—tetracycline; E—erythromycin; CIP—ciprofloxacin; SAM—ampicillin-sulbactam; n.d. = not determined; a–h mark significant differences (*p* < 0.01).

## Sample Availability

Samples of the compounds are available from the authors.
